# Characteristics, motivations and experiences of volunteer befrienders for people with mental illness: a systematic review and narrative synthesis

**DOI:** 10.1186/s12888-018-1960-z

**Published:** 2018-12-04

**Authors:** Sarah Toner, Lauren M. Hickling, Mariana Pinto da Costa, Megan Cassidy, Stefan Priebe

**Affiliations:** 1 Unit for Social and Community Psychiatry (WHO Collaborating Centre for Mental Health Service Development), Queen Mary University of London, London, UK; 2 Institute of Biomedical Sciences Abel Salazar (ICBAS), University of Porto, Porto, Portugal

**Keywords:** Review, Befriending, Characteristics, Mental illness, Volunteering, Motivation, Experience

## Abstract

**Background:**

The literature suggests that many people in the general population tend to distance themselves from those with mental illness. However, there are volunteers that behave differently, spending their free time with people with mental illness and providing direct input in the form of befriending. Whilst there are a range of befriending programmes, little is known about who these volunteer befrienders are, and a previous review of different forms of volunteering in mental health care found data on only 63 befrienders.

**Methods:**

We conducted a systematic electronic search of databases (BNI, CNIL, EMBASE, MEDLINE, PsycINFO, Cochrane Registers, Web of Science) to detect all papers reporting characteristics of befriending volunteers in mental health care published between 2011 and April 2018. The articles retrieved were combined with previous papers identified in an earlier review and with relevant papers identified by experts in the field. The articles that met the inclusion criteria were extracted and narratively synthesised.

**Results:**

Nine studies met the inclusion criteria for this review, reporting characteristics of a total of 577 volunteer befrienders. The most often reported characteristics were age and gender, motivations to volunteer and experience of the role. Whilst characteristics vary greatly, most volunteers are female, and the average age is 50 years. Motivations generally fit into the categories of “giving” and “getting” and experiences are mixed.

**Conclusion:**

Published research on volunteer befrienders has increased in the last eight years, but is still limited. The range of characteristics suggests that there is a potential for encouraging a variety of people to volunteer as befrienders for people with mental illness. Understanding the characteristics and motivations of volunteers may help refine programmes and improve the experience of the volunteer befrienders.

**Electronic supplementary material:**

The online version of this article (10.1186/s12888-018-1960-z) contains supplementary material, which is available to authorized users.

## Background

People with mental illness can often face negative stereotypes, discriminatory behaviour and sometimes even self-stigma [1], and the literature suggests that some people in the general population have a tendency to distance themselves from those with mental illness [2]. Despite this, some people seek direct contact and offer their free time as volunteers to support individuals with mental illness.

A particular form of volunteering in mental health care is befriending [3, 4], where volunteers usually provide repeated, one-to-one and face-to-face contact to develop social relationships with people with mental illness. Some studies reported a reduction of depressive symptoms [5, 6] and social isolation [7] in people supported by befrienders. The literature suggests that volunteer befrienders can reap benefits as well, even outside mental health, such as being able to help others, gain experience, or develop a sense of satisfaction from befriending [8, 9].

Yet, research on befriending is limited, and little is known about the characteristics of those who volunteer to become befrienders [5]. Such information is crucial for befriending programmes as it is imperative to find, recruit and retain the necessary volunteers.

Motivations of volunteers have been explored previously, for example, as illustrated in the ‘Octagon Model’ [10], which covers the dimensions of ‘giving’ versus ‘getting’, ‘distance’ versus ‘proximity’, ‘thought’ versus ‘action’, and ‘continuity’ versus ‘newness’ of experience.

In 2012, Hallett et al. [11] published a systematic review of volunteers in mental health care, addressing their characteristics, reasons for volunteering, and experience in the volunteering role (based on a search in November 2010). However, Hallett et al.’s [11] review included all types of volunteering, such as, helping in groups, providing training in educational programmes, and one-off support for events. It found information on a total of 540 volunteers but only 63 of these were befrienders. Hallett et al. [11] identified a generally mixed demographic; however, a large proportion of befrienders were female. Additionally, experiences of befriending were mostly positive, and the motivations of “giving” something to others or “getting” something from the experience were cited commonly for volunteer befrienders.

Against this background, our aim has been to conduct a systematic review to identify the characteristics of volunteer befrienders and to describe their motivations and experiences. We assumed that the literature since the Hallett et al. [11] review would provide more data on which to draw conclusions about who volunteers to befriend people with mental illness.

## Methods

A systematic literature review, in accordance with the Preferred Reporting Items for Systematic Reviews and Meta-analyses (PRISMA) guidelines [12], was used to identify the characteristics of befriender volunteers in mental health. This was combined with a narrative synthesis grounded on the guidelines developed by Popay et al. [13] to describe their motivations and experiences in volunteering.

### Search strategy

We searched the following databases: BNI, CNIL, EMBASE, MEDLINE, PsycINFO, Cochrane Registers, Web of Science (Psychiatry) and grey literature databases. The following search terms were used: volunteer descriptors (Group 1), mental health descriptors (Group 2) and outcome descriptors (Group 3) (Full list of search terms is available as Additional file 1). The results from this search conducted on 11 April 2018 were included.

A secondary hand search was performed in relevant psychiatric journals, grey literature and references, and experts were contacted to identify additional relevant papers in the field.

### Eligibility criteria

The review included primary studies reporting data on volunteer befrienders. Eligibility criteria were: i) participants were unpaid lay/nonprofessional volunteers, ii) the volunteer activity was a regular commitment (e.g. not a ‘one-off’) with an adult mental health population, iii) the volunteering activity involved only one-to-one, face-to-face contacts.

Texts were excluded if they reported information about: i) volunteers that were currently peers, peer workers, family members, paid carers, paid lay workers, mental health professionals or already known friends, ii) the volunteer activity was not specific to a population with severe mental illness (e.g. dementia/substance abuse/HIV/refugees/cancer volunteering), iii) the volunteering had other formats beyond one-to-one (e.g. groups), face-to-face contact (e.g. telephone helpline/online volunteering), iv) volunteering was part of a course requirement, v) volunteering was a one-off activity (e.g. helping after a natural disaster), vi) the literature was in an inappropriate extraction material (e.g. charity advertising booklet or report).

### Data extraction and analysis

Following the search, all potential studies were exported into EndNote version X5 bibliographic software and duplicates removed. Titles and abstracts were screened for inclusion with a random selection of 20% of the abstracts being screened by a second assessor. If there was any ambiguity about the study, the full paper was obtained and reviewed by two assessors. Inter-reviewer agreement at the screening stage was 94%. There were no discrepancies relating to those papers selected for inclusion in the full-text search. Data extraction was completed by two assessors. Disagreements regarding full-texts were addressed by discussion and input from a third reviewer.

The following quantitative and qualitative data of the studies were extracted: i) about the studies: year, country, setting, aims, and methods; ii) about volunteers: number, age, gender, education level, employment status, religion, ethnicity, relationship status, living arrangements, motivations, previous experience in mental health volunteering, previous connection to organisation, previous experience as a patient in mental health care, volunteer role, volunteer activities, length of commitment, positive and negative experiences; iii) about the volunteering organisation: type, patient group supported, benefits to people with a mental illness, method of recruiting volunteers, volunteer selection criteria, matching process, volunteer training and supervision.

Narrative synthesis was used to analyse the motivations and experiences of the volunteers. MPC independently conducted the preliminary synthesis based on the extracted findings. By deductively conducting the narrative synthesis, preliminary themes were developed based on the hypothetic-deductive approach featured in the literature, with motivations categorised either as ‘giving’ (doing something for others) or ‘getting’, (doing something for themselves); and experiences as ‘positive’ or ‘negative’.

These overarching categories were discussed with all members of the team through an iterative process of continuous discussion, critical reflection, reference to the extracted data and feedback, to ensure a range and depth of the materials. Similar concepts were grouped into the most suitable overarching category, and sub-categories were created where relevant. Tabulation and grouping data was used, selecting suitable illustrative quotations.

## Results

**Fig. 1:**
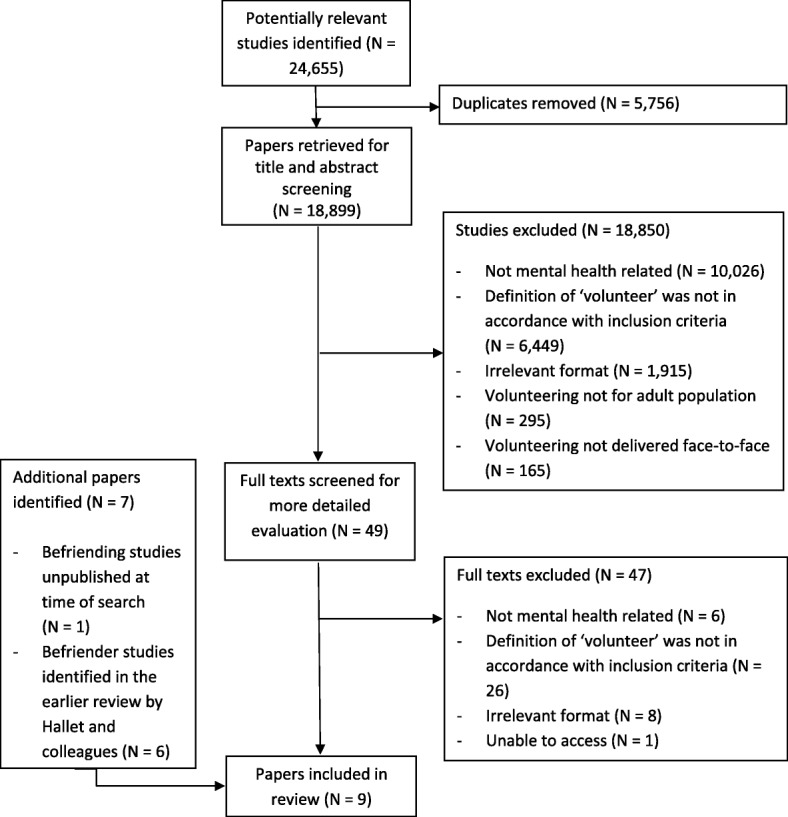
PRISMA Diagram

The searches yielded a total of 24,655 papers which were then screened. Of these, two met the eligibility criteria. Results were combined with the studies identified in the earlier review [11], and one paper identified by experts that was unpublished at the time, but has been published since. A total of nine studies reporting volunteer befriender characteristics were included. The results of the searches and reasons for exclusion are detailed in Fig. 1.

### Overview of the papers

As shown in Table 1, papers identified in the current review came from five different countries: five from the UK [14–18], and one each from Austria [19], Germany [20], Ireland [21] and the USA [22] and included a variety of study designs. Two papers did not state how many volunteers were included, and three provided data on fewer than 30 volunteers [18, 20, 22]. Not including the volunteers in the papers where no numbers were provided, we obtained information on a total of 577 volunteers. Texts were written in English or German.

**Table 1:** Summary of papers included in the systematic review

Country	Year	Study design	Number of volunteers interviewed	Volunteer age (mean and age range)	Volunteer gender (% female)	Mental disorders of patients^b^
Austria [19]	2018	Survey of volunteers across 5 of the 8 regions in Austria	360	54.5	78.8	Adults with Serious Mental Illness (SMI)
^α^Germany [20]	1990	Small questionnaire study	13	21–27	“Mostly female”	Chronically mentally ill
Ireland [21]	2015	Prospective RCT of befriending	73	Not stated	Not stated	Adults with SMI
UK [14]	2013	Service evaluation (questionnaire and qualitative interviews with volunteers)	80, 14 of whom took part in qualitative interviews	20–60 years-old	Not stated	Mothers with post-natal depression and complex needs
^α^UK [15]	1989	Naturalistic study, description of service	31	18–59	73.3	Isolated and lonely users of outpatient psychiatric services. Diagnoses: schizophrenia, manic depressive psychosis, depressive neurosis, anxiety states, dependent personality disorder.
^α^UK [16]	2003	Naturalistic study, profile of service	No detail	No detail	No detail	Socially isolated outpatients experiencing long standing mental health problems. 36% have depression, 10% dual diagnosis, 54% other (schizophrenia, manic-depression, anxiety, and long-term mental health problems).
^α^UK [17]	2003	Naturalistic study, profile of service	No detail	University students	Not stated (‘Problems recruiting enough male volunteers’)	People with enduring or severe/complex mental health problems. 70% of the 450 had schizophrenia.
^α^UK [18]	2011	Small questionnaire study	8	50 (29–65)	25	Adults (outpatients) with difficulties to form and sustain friendships due to moderate/severe mental health problems.
^α^USA [22]	2009	Naturalistic study, service evaluation	12	Unclear. All but one estimated to be over 30, some of retirement age	66.7	People with severe mental illness (outpatients). Specific psychiatric diagnosis not obtained.

^a^Papers taken from review conducted by Hallett et al. [11]

^b^Using the terminology of the original publications

Total befrienders (N) = 577 (360 + 13 + 73 + 80 + 31 + 8 + 12)

### Volunteering programmes

The majority of befriending programmes were run by third sector, non-profit organisations [14–20, 22], and one was specifically set up for a randomised controlled trial (RCT) run by an Irish university [21]. Many studies stated that the aim of befriending was to reduce social isolation and promote social activities and inclusion [14, 16, 18].

Six papers mentioned the duration of the befriending relationship. For most programmes, the intended average duration was 12 months with large variations of the actual relationship length [14, 16–18, 22]. One programme requested a nine month commitment from befrienders [21].

Three studies referred to matching befrienders and befriending recipients (hereafter, referred to as befriendees). Sheridan et al. [21] reported that their volunteer befriender cohort completed a profile of demographic characteristics, social life and leisure preferences. This was used to match participants based on shared interests. Similar approaches were taken in the remaining two studies [17, 18].

Training and supervision of befrienders were outlined in six papers, in which both were described as compulsory [14–18, 22]. This ranged from a one-day programme [21] to six days of training delivered by the project co-ordinator, covering anticipated roles and responsibilities [14]. In another programme befrienders received support during the programme when needed but no initial training [20].

The processes of recruiting befrienders were described in three papers. Sheridan et al. [21] recruited them for their RCT through an “extensive strategy” across a wide range of groups and organisations in the community. Among the remaining studies [16, 17], newspaper advertisements were most often used followed by poster displays, word of mouth, local radio adverts, leaflets and student enquiries.

Selection processes for choosing befrienders were discussed by two papers. For the RCT [21], befrienders were selected for participation through an interview process, completion of a police vetting process and two character references for each application. The other study recruited “current psychology undergraduates or graduates who have expressed a desire to do clinical psychology training” [17].

### Characteristics of volunteers

#### Age

As shown in Table 1, seven papers provided some information on the age of volunteers. Befrienders had a mean of 50 years-old with a very wide range between 18 and 65 years old [14, 15, 18–20, 22].

#### Gender

Six of the papers reported the gender of the befrienders. In most papers, the majority of befrienders were female [15, 17, 19, 20, 22]. Only one study [18] reported a sample with more males (75%) than females.

#### Employment status

Two papers indicated the employment status of the volunteers. One reported that half of volunteers were retired [18] with the remainder either unemployed or in full-time education. Another study reported that all volunteer befrienders were university students [17].

#### Ethnicity

The ethnicity of the volunteers was reported in one paper from the United Kingdom. The sample was described as ‘mainly White British’ [14].

#### Relationship status

The relationship status was included in three papers. Two of these found that the majority of volunteers were married or in civil partnerships [14, 19]. In contrast, the majority (10/13) of volunteers described by Brackhane and colleagues [20] were living alone.

#### Past psychiatric history

Two papers gave information about the volunteers’ past psychiatric history, reporting that approximately one third of befrienders had personal experience of mental health care [18, 22].

#### Motivations for volunteering

Four papers [14, 15, 17, 19] reported the motivations of the befrienders in the study (Table 2). Motivations to volunteer were studied by Klug and colleagues [19], using a list of nineteen predefined motivations.

**Table 2:** *Motivations of volunteer befrienders*

Getting	
Tombs et al., 2003 [17]	*“undergraduates and graduates enquiring about shadowing or unpaid placements in order to gain experience for clinical training”*
	*“to gain assistant and research assistant post”*
Klug et al., 2018 [19]	*“Curious to find out if I am suitable for the role”*
	*“Test out career aspirations”*
	*“Enhance my awareness of mental health issues”*
	*“Befriending looks good on my CV”*
	*“To gain psychologically relevant experience (for my career)”*
	*“Acquire new skills”*
	*“Meet new people”*
	*“Find explanations for my own behaviour”*
	*“Have close contact with others”*
	*“To feel like a better person”*
	*“To feel needed and acknowledged”*
	*“To be accepted and liked”*
Giving	
Kingdon et al., 1989 [15]	*“a practical way of giving something back after being helped”*
Coe et al., 2013 [14]	*“I wanted to give something back to the community really and I feel that I have done that. Um. It’s kind of made me feel accepted in a way”*
Klug et al., 2018 [19]	*“I have received voluntary help in the past, and wanted to give something back”*
	*“Feel responsibility to help others”*
	*“Helping others is part of my philosophy of life”*
	*“Helping others is part of my religious belief”*
	*“I wanted to do something useful with my spare time”*

The different reasons and motivations of volunteers were categorised in two main themes, “getting” and “giving”, as illustrated in Table 2.

##### “Giving”

These motivations were expressed in four studies [14–16, 19], where volunteers shared a desire to give something back through the programme. Kingdon et al. [15] stated that their befrienders wanted to take part in a befriending programme to give back to the service that they had used previously. Similarly, many volunteers working with mothers with post-natal depression had experienced post-natal depression themselves, and described this as a motivation for volunteering [14]. McGowan et al. [16] reported that volunteer befrienders wanted to “give” something of themselves during their time, although they did not describe more specific examples.

##### “Getting”

Five studies described “getting” as a motivation for volunteers [14, 16, 17, 19, 20]. Still, what volunteers wanted to get was quite varied, and ranged from: i) ‘getting involved with the community’ [14], ii) testing out their own suitability for befriending role, iii) finding explanations for their own behaviours [16, 20], iv) testing out potential career aspirations [16, 17], v) learning more about local mental health services, vi) acquiring new skills or vii) meeting new people [16].

#### Experiences of volunteering

Volunteers’ experiences in the role were discussed in five of the ten papers [14, 17, 18, 20, 22]. The experiences described by the volunteer befrienders were quite varied, at times positive (Table 3) and on other occasions negative (Table 4).

**Table 3:** *Positive experiences of the volunteer befrienders*

1.Satisfaction with the relationship with the befriendee	
1.1.Spending nice time together	
McCorkle et al., 2009 [22]	*“meeting each other’s families, dining in each other’s homes, celebrating holidays together.”*
	*“He enjoys getting together. We enjoy each other, getting together and talking, and I’ve decided that that’s of value to me.”*
1.2.Trusting each other	
Mitchell & Pistrang, 2011 [18]	*“She seems to be able to talk to me about all sorts of things. Sometimes really personal things… it’s a confidential situation, it’s not going any further than us. So maybe that’s what gives her the freedom to talk.”*
	*“While I’m talking to him I’m not constantly thinking of the roles that I’m the befriender and he is the befriendee, we’re two people having a chat.”*
	*“I’m just myself and he’s just himself, we just happen to be doing this particular thing, in this particular relationship, in this particular way*. *.*. *It’s more important for us just to be ourselves.”*
McCorkle et al., 2009 [22]	“We’re there for each other.”
1.3. Wanting to continue the relationship as friends	
McCorkle et al., 2009 [22]	*“If [the befriending scheme] ended, he and I would probably still be friends 10 years hence, still doing some stuff together”*
	*“in this movement from ‘helper/helpee’ to true friends”*
2. Good experience with the volunteering scheme	
2.1.Access to support/supervision	
Tombs et al. 2003 [17]	*“the most useful aspect being the provision of supervision by clinical psychologists and advice about writing application forms”*
Mitchell & Pistrang, 2011 [18]	*“When she was cutting it was really difficult and I was really distressed about it, so I called [befriending scheme coordinator] to see how to handle it…so it was like dealing with it together. It’s not like I’m alone dealing with the situation.*”
2.2.Usefulness of sharing experiences with other volunteers	
McCorkle et al., 2009 [22]	*“It’s so nice because other volunteers who’ve already gone through it and have found out what works have helped me a lot.”*
3. Personal gains with the relationship	
3.1. Feeling good to provide new experiences to the befriendee	
Mitchell & Pistrang, 2011 [18]	*“to get out and visit places and do things that otherwise [befriendee] wouldn’t have done naturally on his own, and that’s an exposure to a whole load of different things…it’s opening that window of things out there.”*
	*“about creating opportunities for [befriendee] to go where perhaps he wouldn’t have gone before in relationships.”*
3.2. Filling their own free time	
McCorkle et al., 2009 [22]	“filling the gap created by retirement.”
3.3. Feeling rewarded for contributing to the befriendee’s recovery	
McCorkle et al., 2009 [22]	*“No matter how much time, or lost sleep, or stress you feel the investment requires, the satisfaction of being intimately involved with another life in recovery is just extraordinarily self-enhancing, reinforcing.”*
	*“I feel good about myself that I’ve been able to do something for him.”*
Coe et al., 2013 [14]	*“But I remember this particular girl the first time I met her she just … I could tell by her eyes what pain she was in. She just had … she sort of glared at me. And now she does actually look happy again and there is that sparkle in her eyes.”*
	*“It’s just really … I just found it really rewarding. I wanted to give something back to the community really and I feel that I have done that. Um. It’s kind of made me feel accepted in a way.”*
3.4. Being supported by the befriendee	
McCorkle et al., 2009 [22]	*“I like it that she’s been there even for me, when I needed someone to lean on, that I could talk to her.”*
3.5. Learning/reflecting about themselves	
McCorkle et al., 2009 [22]	*“required dealing with one’s own negative preconceptions about mental illness.”*
Mitchell & Pistrang, 2011 [18]	*“…it’s part of that looking at whatever the situation is, from a lot of different perspectives…You look at it in a balanced type of way, rather than in one fixed way.”*
	*“It makes me think about me, who I am…you do have to say to yourself, ‘Am I happy with where I am?’, and if there are things that are getting to me where is that layer occurring and you know, because I do become more conscious.”*
	*“It helps you reassess some of the things that have happened to yourself, and how other people may have reacted or looked at it.”*
3.6. Changing attitudes towards people with mental disorders	
Mitchell & Pistrang, 2011 [18]	*“… It’s nice to sort of confirm that what you read in the papers isn’t representative of the mental health.”*
4. Professional gains with the experience	
4.1. Having contact with people with mental disorders	
Mitchell & Pistrang, 2011 [18]	*“I don’t know anyone with a diagnosed mental disorder so I had no idea what someone like that would be like.”*
4.2. Helping to clarify their career path	
Tombs et al., 2003 [17]	*“it has also been useful in clarifying whether clinical psychology is the career.”*
4.3. Helping to build the CV	
Tombs et al., 2003 [17]	“*[helped with the] demand for relevant voluntary experience whilst competition for assistants’ posts remains high and most posts require some previous client experience.”*

**Table 4:** *Negative experiences of the volunteer befrienders*

1. Bad experience with the volunteering schemes	
1.1.Bureaucracy/waiting when recruited	
Tombs et al., 2003 [17]	*“the increase in delays in registering befrienders might well have a negative impact on recruitment as volunteers may need to wait for up to six months before being allocated a client.”*
	*“waiting three months for clearance to proceed and, regrettably … may have to wait several months more.”*
1.2.The costs linked with the activities	
Tombs et al., 2003 [17]	*“volunteers are expected to pay for their own refreshments and entertainment.”*
McCorkle et al., 2009 [22]	*“insisted on full equality of financial contribution and decision making so that the [befriending] relationship did not encourage passivity and dependency.”*
1.3. Feeling pressured with the commitment to meet	
Brackhane et al., 1990 [20]	*“tension between free voluntary input and a sense of duty or obligation”*
McCorkle et al., 2009 [22]	*“after a rough day at work, meeting could feel more like a commitment than like fun.”*
2.Dissatisfaction with the relationship with their befriendee	
2.1.Expectations of their befriendee not being met	
McCorkle et al., 2009 [22]	*“expected a client who was much younger, physically active, and interested in going places and doing things, but ended up with a middle-aged client without those interests.”*
2.2.Disliking their befriendee	
Tombs et al., 2003 [17]	*“Declined befriending … because they did not like the potential befrienders on offer”*
McCorkle et al., 2009 [22]	*“Then I got to thinking, not every match is going to succeed and go off and go to college.”*
2.3.Difficult to empathise with the befriendee	
Mitchell & Pistrang, 2011 [18]	*“Some of it I can empathise with and some of it I’ve absolutely no idea at all…I don’t think you will ever get a hundred percent fit with other people… And if you did have that hundred percent fit, it might be ideally the wrong person for them because they’ll just wallow in it with them.”*
3. Challenges in the relationship	
3.1.Difficulties in adopting an attentive/supportive role as a volunteer	
Brackhane et al., 1990 [20]	*“avoided emotional talks because of anxiety to get too much worked up about it”*
Mitchell & Pistrang, 2011 [18]	*“I’d say the hardest thing is not giving a true reaction to the things she says, and biting my lip rather than making or voicing my judgments or opinions…”*
	*“I’m keeping a watchful eye, but not making it obvious”*
3.2.Difficulties in setting boundaries	
Mitchell & Pistrang, 2011 [18]	*“It’s more to do with where I’m putting my boundaries… It’s kind of making sure that the whole conversation isn’t about me…The unequal-ness of the relationship is that one. It’s not about me.”*
3.3.Difficulties in dealing with confidentiality/privacy	
McCorkle et al., 2009 [22]	*“the awkwardness of running into a friend or business acquaintance when with one’s match.”*
3.4.Difficulties in tolerating the befriendee’s behaviour	
McCorkle et al., 2009 [22]	“[tolerating] heavy smoking and coffee consumption, occasional outbursts of anger.”
3.5.Feeling exploited by the befriendee	
McCorkle et al., 2009 [22]	*“There was no joy in it for me by going and picking her up and taking her to whatever store she wanted to go to. I knew I wouldn’t last like that. So I started setting limits and explaining to her that that’s not what friends do. They do that occasionally, maybe, but that isn’t what a friend does.”*
	*“feeling treated as a taxicab was an unpleasant experience.”*
3.6.Difficulties in ending the relationship	
McCorkle et al., 2009 [22]	*“…He’s as far as he’s gonna be, I think, but I still can’t leave him, ‘cause I feel like we’ve just developed a bond!”*
	*“I can’t imagine not having my [befriendee] friend in my life. I really can’t”*
	*“worried about what would happen if life changes (such as moving elsewhere for graduate school) prevented continuation of the relationship.”*
Mitchell & Pistrang, 2011 [18]	“*I feel like it’s slightly kind of a bit like a taboo subject [the end of the relationship]. Um, I think I would be scared of saying the wrong thing, if it came up.*”

##### Positive experiences

Qualitative interviews from Coe and Barlow [14] portrayed positive experiences of volunteer befrienders, describing ways in which they supported the befriendee, feeling rewarded for contributing to the befriendee’s recovery. The positive experience of feeling good about helping someone else was also expressed by eight of 12 volunteers interviewed by McCorkle and colleagues [22]. Some described ways in which volunteering contributed to them personally, such as increasing their self-esteem, confidence and acceptance [14]. Many volunteers felt that with time they gained a genuine friend themselves, wanting to continue the relationship as friends, with many expressing that the benefits of one-to-one volunteering far outweighed the cost in time, money and energy [22].

A positive impact from volunteering on attitudes toward mental illness was discussed in three papers [18, 20, 22]. The experience of volunteering made befrienders reevaluate their preconceptions surrounding mental illness, which they considered a valuable growth opportunity [22]. In some cases this changed previously negative attitudes toward people with mental illness; some lost their initial concerns about the unreliability of people with a mental illness, and found them “surprisingly normal” [20]. For others with personal experience of mental illness, volunteering offered a new perspective on their own problems [18], learning and reflecting about themselves, and instilled a sense that they had benefitted from befriending as much as the befriendee [22].

##### Negative experiences

Negative experiences were described in relation to the behavior of the befriendee in three papers [18, 20, 22]. Some befrienders were left feeling unappreciated [22] when the befriendee did not maintain communication, was often late, or appeared to use the befriender “as a taxicab”. Other volunteers expressed difficulties in dealing with confidentiality and privacy, balancing their personal feelings with a sense of professionalism when sensitive information was disclosed by the befriendee [18]. Another matter which was disliked was the recruitment process [17, 21], the bureaucracy (police checks) and waiting (lengthy delays) experiences once recruited, making befrienders drop out of the programme before they had even begun.

## Discussion

### Main findings

This review provided data on 577 volunteer befrienders from nine studies, around nine times as many befrienders as summarised in a previous review. Yet, more than half of the total number of included befrienders derived from one large study in Austria [19].

The findings suggest that the mean age of befrienders is 50 years-old, but with a very broad range, that they are predominantly female, and that many have experience of psychiatric treatment either themselves or through a close friend or family member. Overall, there was a substantial variation in the socio-demographic characteristics of befrienders between, and also within, studies.

In terms of motivations for befriending, both “giving” something to the befriendee community or in general, and “getting” something out of the programme for themselves appear important.

Positive experiences generally centre around the impact of befriending on the befriendee and own changes in attitudes toward mental illness, while negative experiences related to feeling unappreciated by the befriendee or bad experiences with the befriending organisation. Volunteers had mixed feelings about their relationship with paid mental health professionals and tend to perceive a lack of clarity surrounding their role as a befriender.

### Strengths and limitations

To our knowledge, this is the first systematic review focusing on the characteristics, motivations and experiences of befrienders in mental health care. It provides a summary of the different motivations and experiences of befriender volunteers that appear plausible and are in line with previous literature on volunteering. It covers a significant number of befrienders, but also a range of countries, and languages (English and German) exceeding those in a previous wider review on different types of volunteering in mental health care [11]. A further strength is that to reduce the publication bias, unpublished literature and hand searches of relevant journals were performed to maximise the search. In addition, the themes of the narrative synthesis were identified across a diversity of papers and were discussed within the multidisciplinary team of the co-authors to reduce bias of findings.

The study, however, has several limitations. First, whilst the total number of 577 included befrienders may allow more general conclusions than previously possible, it is still small in the light of the wide use of befriending schemes in some countries, particularly considering that data on 360 befrienders were from only one large survey [19]. Secondly, all papers are from a limited number of high-income countries, and there remains a lack of information about what types of befriending programmes, if any, exist in other countries and what the characteristics of the befrienders in those programmes are. Thirdly, although it was possible to extract some qualitative data about the motivations and experiences of volunteers, papers included only a few quotations, and hence the available data was limited to conduct the narrative synthesis. Fourthly, the quantitative data extracted for this review were not consistently reported, which makes it difficult to synthesise the data and compare studies. Finally, with respect to experiences, one may assume that most studies were biased through the selection of interviewees. Befrienders with very negative experiences are more likely to end their befriending sooner and avoid participation in research interviews than those who liked their befriending role and did it for longer.

### Comparison with the literature

Whilst the earlier wider review [11] identified details on only 63 volunteer befrienders, this review found reports on an additional 514 befrienders. Given that the previous review covered the whole period of time since the inception of the included databases and this one added only eight years, the increase of research data on befrienders may reflect a rising research interest in befriending programmes.

The findings here are consistent with those reported by Hallett and colleagues [11]. This is in regards to the predominance of women among befrienders, the otherwise wide range of socio-demographic characteristics, the mix of motivations of “giving” and “getting” for volunteer befrienders, and the mostly positive but still mixed experiences of befrienders.

The categorisation of motivations into “giving” and “getting” reflects one of the four dimensions of the ‘Octagon Model’ which has been used to describe the motivations of volunteers in general [10]. Klug and colleagues [19] applied such categorisations in their assessment of volunteer motivations. They identified four subgroups of befrienders based on their characteristics (age, gender, marital status and employment status), which were associated with different motivations to engage in befriending. The largest subgroup included married, retired females (median age = 64), which were largely motivated by their responsibility to help others. This motivation was also predominant in two other subgroups, comprised of married retired males (median age = 58), and married females in full-time employment (median age = 51). The final subgroup consisted of single females in full-time employment (median age = 44). The most frequent motivations in the four groups were similar and included aspects of both ‘giving’ and ‘getting’. Only the fourth and youngest group differed in some aspects from the other groups and stated more often the wish to acquire new skills and career aspirations as motivations. It remains unclear to what extent different subgroups of befrienders may respond to different recruitment strategies and benefit from different types of programmes and supervision.

### Implications

The fact that there is a wide variation in the characteristics of volunteer befrienders, including employment and relationship status, suggests that a typical volunteer befriender does not exist and that there is a potential to recruit volunteers for befriending programmes from very different groups in the population. The majority of befrienders being female might reflect gender differences in attitudes, social roles or both. The motivations of different groups and their potentials to engage in specific befriending programmes are likely to vary, and more research is required to tailor recruitment strategies and programmes for people with different backgrounds, interests and social situations. In particular, additional qualitative research should be conducted to further explore the motivations and experiences of different volunteers. Studies with sufficiently large samples should allow the exploration of potentially relevant subgroups, of which only one study has been able to do so far [19].

Allowing volunteering opportunities that are consistent with volunteers’ motivations may improve volunteers’ satisfaction and retention in these programmes. Equally, exploring the experiences in more detail may help with designing programmes that maximise the satisfaction of befrienders and encourage them to stay in programmes or volunteer again if they had engaged previously and since ceased participating.

Whilst this review summarised data of research publications, this is still limited and inconsistently reported. Therefore, in the future, data about volunteers should be routinely collected and published on a large scale to map out and further develop befriending programmes. This is currently hindered by a number of factors, one of which being that many programmes are run outside mainstream health services with less developed data documentation systems.

## Conclusions

The results reported in this review suggest an increased attention to befriending programmes in research over the last eight years. Apart from most befrienders being female, the variability of characteristics of befrienders suggests that generalised conclusions are difficult. At the same time, it may underline the wide potential to establish more befriending programmes in different contexts, whilst the different motivations and experiences of befrienders point to possibilities for refining and specifying programmes for different types of befrienders.

## Additional file

Additional file 1: Characteristics, motivations and experiences of volunteer befrienders for people with mental illness: A systematic review and narrative synthesis. (DOCX 22 kb)

## Abbreviations

NHS: National Health Service
NIHR: National Institute of Health Research
PRISMA: Preferred Reporting Items for Systematic Reviews and Meta-Analyses
RCT: Randomised Controlled Trial
SMI: Serious Mental Illness
