# Supplementary material for: Characteristics, motivations and experiences of volunteer befrienders for people with mental illness: a systematic review and narrative synthesis
Source: BMC Psychiatry. 2018 Dec 4;18:378. doi: 10.1186/s12888-018-1960-z (PMC6278150; doi:10.1186/s12888-018-1960-z)
Supplement: Supplementary file 1 — Characteristics, motivations and experiences of volunteer befrienders for people with mental illness: A systematic review and narrative synthesis. (DOCX 22 kb) [file 12888_2018_1960_MOESM1_ESM.docx]

**Characteristics, motivations and experiences of volunteer befrienders for people with mental illness: A systematic review and narrative synthesis**

Search Terms

*Group 1: Volunteer Descriptors*

volunteer* OR lay helper*OR befriend*OR companion OR friend OR compeer OR peer OR buddy OR unpaid carer OR informal caregiver OR voluntary caregiver OR naturalistic contact OR supported socialisation OR psychosocial support OR supported friendship OR peer assistance OR intentional friendship OR consumer run services OR consumers as providers OR consumers-as-providers OR community support OR community services OR paraprofessional* OR nonprofessional volunteer* OR nonprofessional worker* OR citizen participation OR civic participation

*Group 2: Mental Health Descriptors*

mental health OR mental illness OR mental problem OR mental disorder OR mental health scheme OR mental health charity OR mental health project OR mental health program* OR mental health organisation OR mental health service OR mental health care OR psychiatry OR psychiatric scheme OR psychiatric charity OR psychiatric project OR psychiatric program* OR psychiatric organisation OR psychiatric service OR psychiatric care OR psychosis OR schizophrenia OR severe mental illness OR drug and alcohol OR depression

*Group 3: Outcome Descriptors*

motivation* OR motive* OR reason* OR opinion* OR attitude* OR experience* OR reward* OR benefit* OR success* OR drawback* OR negative* OR failure* OR challenge* OR difficult*
